# Managing clinically significant findings in research: the UK10K example

**DOI:** 10.1038/ejhg.2013.290

**Published:** 2014-01-15

**Authors:** Jane Kaye, Matthew Hurles, Heather Griffin, Jasote Grewal, Martin Bobrow, Nic Timpson, Carol Smee, Patrick Bolton, Richard Durbin, Stephanie Dyke, David Fitzpatrick, Karen Kennedy, Alastair Kent, Dawn Muddyman, Francesco Muntoni, Lucy F Raymond, Robert Semple, Tim Spector, UK 10K

**Affiliations:** 1 Nuffield Department of Population Health, HeLEX – Centre for Health, Law and Emerging Technologies, University of Oxford, Oxford, UK; 2 The Wellcome Trust Sanger Institute, Wellcome Trust Genome Campus, Hinxton, Cambridge, UK; 3 CELLS – Centre for Ethics and Law in the Life Sciences, Institute of Philosophy, Leibniz Universitaet Hannover, Hannover, Germany; 4 Department of Medical Genetics, Cambridge Institute for Medical Research, University of Cambridge, Cambridge, UK; 5 MRC CAiTE Centre, School of Social and Community Medicine, University of Bristol, Oakfield House, Clifton, Bristol, UK; 6 Institute of Psychiatry, Kings College London, London, UK; 7 MRC Institute of Genetic and Molecular Medicine, University of Edinburgh, Western General Hospital, Edinburgh, UK; 8 Genetic Alliance UK, London, UK; 9 Dubowitz Neuromuscular Centre, UCL Institute of Child Health, London, UK; 10 Department of Twin Research, King's College London, St Thomas' Hospital, London, UK; 11 www.uk10k.org.uk

**Keywords:** incidental findings, ethics, sequencing, research, consortia, management pathway

## Abstract

Recent advances in sequencing technology allow data on the human genome to be generated more quickly and in greater detail than ever before. Such detail includes findings that may be of significance to the health of the research participant involved. Although research studies generally do not feed back information on clinically significant findings (CSFs) to participants, this stance is increasingly being questioned. There may be difficulties and risks in feeding clinically significant information back to research participants, however, the UK10K consortium sought to address these by creating a detailed management pathway. This was not intended to create any obligation upon the researchers to feed back any CSFs they discovered. Instead, it provides a mechanism to ensure that any such findings can be passed on to the participant where appropriate. This paper describes this mechanism and the specific criteria, which must be fulfilled in order for a finding and participant to qualify for feedback. This mechanism could be used by future research consortia, and may also assist in the development of sound principles for dealing with CSFs.

## Introduction

Advances in sequencing technology are enabling the rapid generation of information on the whole genome of individuals at an ever decreasing cost. The possibility of identifying clinically significant findings (CSFs) – findings that have potential health or reproductive significance for the individual participant – is greatly increased when whole-genome information is generated.^1^ This increase, coupled with our increased understanding of the relationships between genotype and phenotype, has created a more pressing debate about the release of individual results to research participants. Although there is still considerable diversity of opinion, there is a growing consensus in the bioethics community that, in some contexts, it would be ethical to report back CSFs where they could be of benefit to the participant.^2, 3^ A recent report by the American College of Medical Genetics and Genomics recommended the feedback of a given list of certain variations within the context of clinical genetic testing, but explicitly excluded research studies from their recommendations.^4^ In addition, recent studies have shown that research participants in the United Kingdom and elsewhere^5, 6, 7^ would like to be informed of CSFs made in the course of research.^8^ As yet, there is no widely accepted agreement on what governance procedures should look like within genomic research, although a number of models have been proposed^9, 10, 11, 12^ and exist outside of the United Kingdom.^13^

The UK10K project management framework has been used to feedback CSFs where the research participants involved are recruited either for study of disease, or for general research, without specific disease-related questions to answer. Within this management framework, CSFs are broken down into two categories. These are ‘pertinent findings' (PFs), which relate to the disease under investigation in a particular study within the UK10K project and ‘incidental findings' (IFs).^14^ IFs relate to discoveries outside of the original research objectives of the particular study for which that subject was recruited into UK10K, and were unforeseen at the point at which the participant consented to take part. Researchers in the UK10K study have no obligation to pass IFs on to participants in the study. The reasons for not doing so are because (i) the data quality and inference (predictive ability) is low, (ii) there is a need to verify all findings before action, (iii) it is not possible to really give informed consent for IFs and (iv) the costs and resources involved in providing genetic counselling if all potential IFs were followed up. The UK10K model provides a mechanism for allowing CSFs from PFs to be reported back to patients in a responsible manner within the lifetime of the UK10K project. If a researcher identifies a CSF, the UK10K model allows them to pass their finding on, so that they can be used for clinical care following an established governance framework.

This management pathway was developed by the Ethics Advisory Group (EAG) in consultation with other members of the project and is further outlined in a policy document.^15^ The EAG was formed at the commencement of the project to address any ethical issues that were likely to arise, and included researchers, clinicians, bioethics and legal experts, and patient and cohort participant representatives. The purpose of this paper is to describe the UK10K management framework so that it may assist other projects, which may face similar issues and in doing so further the policy debate about how best to deal with CSFs in genomics research.

## The UK10K project

The UK10K project (2010–2013), funded by a Wellcome Trust Strategic Award, has analysed the DNA of ∼10 000 people to improve the understanding of the role of low-frequency and rare genetic variants in health and disease.^14^ Four thousand participants were drawn from two cohort studies in the UK – TwinsUK and the Avon Longitudinal Study of Parents and Children (ALSPAC), with the remaining 6000 samples being taken from participants in 11 different condition or disease-specific studies. Samples were from the following three sample groups: neurodevelopment (schizophrenia, bipolar, autism (Tampere and Atypical), learning disabilities, Asperger syndrome; obesity; rare diseases (neuromuscular; ocular coloboma; congenital heart disease; ciliopathies disorders; severe intellectual disabilities; congenital hypothyroidism; resistance to thyroid hormone; familial hypercholesterolemia). Many of these participants have an on-going relationship with the recruiting researchers. Where conditions relating to data type and participant standpoint/motivation are appropriate (usually where research participants are also patients and therefore are likely to benefit directly from the anticipated findings of the study) a system of informed consent and information feedback developed along existing lines of clinical interaction has been put in place and will be discussed below.

The sequencing for the UK10K project was largely carried out through the Wellcome Trust Sanger Institute. Four thousand of the participants (TwinsUK and ALSPAC cohorts) were sequenced to approximately sixfold coverage of their entire genome, to provide a detailed sequence reference database connected to phenotypic and clinical data.^14^ As these are unselected participants for the purposes of the study, they do not have an ongoing clinical relationship with any of the researchers in the study, but they do have an ongoing relationship with the custodians of the cohorts. The framework below will therefore primarily relate to the 6000 participants from the disease-specific studies, but not to those from the TwinsUK and ALSPAC cohorts, where different policies apply. For TwinsUK, there is a longstanding policy of returning clinical or laboratory results to the participants and their general practitioners that would be of clinical use to the local doctor in management decisions. Consent and ethical approval for the UK10K study was given on the basis that TwinUK participants would not be provided with the results of the genotypes or sequence variants other than for determining twin status. For genetic data of the type collected within the UK10K project, ALSPAC operate (according to their process of informed consent) a policy of non-disclosure relating to personal data and research findings. Participants are asked explicitly for their agreement to the type of data being collected and to this mode of management. All research findings are communicated only at the level of the population through conventional routes of dissemination.

Sequencing of exomes belonging to the 6000 participants from the disease-specific cohorts to approximately 60 times coverage was expected to identify roughly 20 000 gene variants in each genome.^14^ These participants have medical conditions with a likely underlying monogenic cause and have already consented to genetic research. The aim of the sequencing was to distinguish those genetic variants that may be linked to a particular disease and those having no discernible effect.^14^ This comprehensive analysis of the protein-coding sequences within their genomes, coupled with findings obtained from the first group (TWINS and ALSPAC cohorts), enables the identification of potential causative genetic mutations in the particular diseases observed.^14^ This comprehensive sequencing also raised the possibility of IFs for this group of participants, but as it was carried out in a research setting these CSFs were not of a clinical diagnostic standard.

The reference data set created by the UK10K project is accessible by the wider research community via the European Genome-Phenome Archive (EGA) through a managed-access mechanism. This raises the possibility of researchers outside UK10K also identifying CSFs in the data to which they have been granted access. This raises the additional question of how far the procedure for returning CSFs should extend. Given the range of expertise and specialisms of those external researchers who might wish to access the data from UK10K, the possibility that they would identify further CSFs was very real. The management pathway for handling findings and potential feedback in the case of PFs was not to impose an obligation on third-party researchers, but merely to provide a guiding mechanism to deal with such an eventuality in a responsible manner should it arise. This management framework will only be effective during the lifetime of the project, however, it has been suggested that this approach, or the strategy developed, may be of use to the wider research community. This is of particular relevance in relation to the public release of UK10K data and thus the possibility of researchers outside the project (or study custodians) also identifying CSFs in the data to which they have been granted access.

## The UK10K management framework

The purpose of this management framework is to keep clear the distinction between clinical and research activities and the ensuing obligations to participants, while at the same time allowing the possibility for CSFs to be reported back to patients in a responsible manner where appropriate. When a researcher encounters a CSF, the management framework can be activated. The clinician with a pre-existing relationship with the particular research participant has a key role in the feedback process. Given the large and far-reaching scope of this project, there are four conditions, which must be met in order for the framework to be activated. Only if all of these criteria are met, can the PF or IF be passed on to the participant. These are as follows:

1. To respect the research participant's right to know or, alternatively, their right not to know about CSFs. Explicit consent must have always been given by the participant for IFs and/or PFs to be returned.
2. CSFs should only be returned where they are of significant clinical importance.
3. CSFs should be clinically and analytically validated before being returned to participants by their treating clinician, via a genetically accredited laboratory.
4. Feedback to individual participants should be conducted by a trained professional able to provide genetic counselling.

If one or more of these criteria are absent, information on the CSF will not be fed back to the participant. These are therefore of crucial importance and must be considered before the participant is contacted.

**Figure 1:**
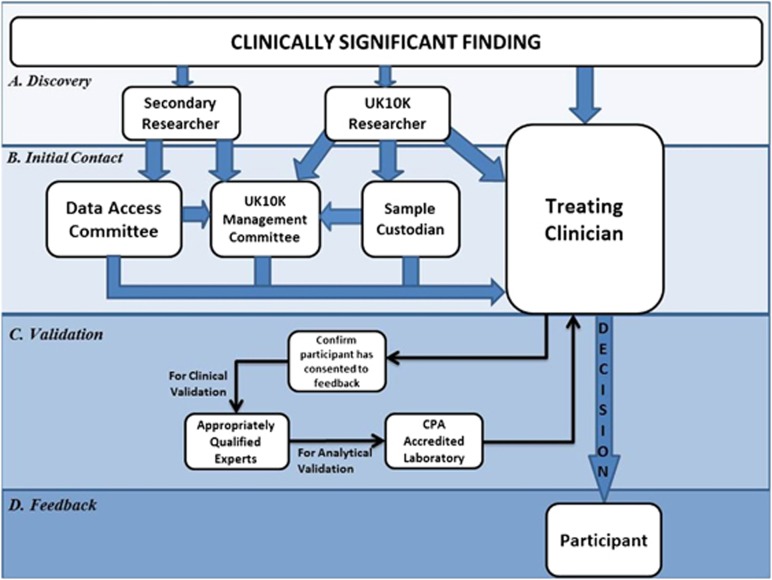
Depiction of the UK10K Management Framework, consisting of four stages.

The UK10K management framework comprises four different stages, from the discovery of a CSF to the return of that finding to the participant. Although the treating clinician has a central role and retains ultimate responsibility for communicating any CSFs to the participant, the particular pathway taken to arrive at this point will depend on the circumstances and whether the person who identifies the CSF is within the UK10K project, or outside of it. Figure 1 below illustrates the four stages within the management framework, and the specific pathways to be taken by researchers in UK10K. It also details the pathway for non-UK10K secondary researchers if they wish to inform the project upon discovering a CSF.

### Different pathways

#### Discovery

There are three types of researcher who might discover CSFs in the UK10K data:

1. Treating clinicians are UK10K clinical researchers who have an ongoing clinical role in relation to that participant. They may have recruited participants into the study and may be the sample custodian for previously collected samples or those collected within the lifetime of the project.
2. UK10K researchers who carry no clinical role in relation to the participant in question, which may include clinician scientists; and
3. Secondary researchers accessing UK10K data through the EGA.

One of the fundamental principles in developing this framework was that CSFs could only be returned by the clinician who has a pre-existing relationship with the participant. As a result, they are a key player within the mechanism. Responsibility lies with the treating clinician in liaising with participants, and determining whether any findings should be reported back to them. They also determine whether it would be feasible to report back CSFs. All consent forms and management pathways for feeding back CSFs are approved by a research ethics committee. All UK10K researchers should share PFs with other UK10K researchers as part of that research collaboration, but the participant will not necessarily receive this information. Secondary researchers are those who have accessed UK10K data in the EGA via the managed data access mechanisms for their own independent research, and have no role in the feedback process once they have informed the project of their finding.

#### Initial contact

On discovery of a CSF, the management pathway for each type of researcher is as follows:

1. If the CSF is identified by a UK10K researcher, they should first contact the relevant Sample Custodian (who may also be the treating clinician) or the UK10K Management Committee. The UK10K project Management Committee can then ensure that findings are passed on to the relevant treating clinician responsible for that participant, and the management framework is activated.
2. If a secondary researcher decides to activate the management framework, they should contact the UK10K Management Committee or the Data Access Committee, who will then pass the findings on to the relevant Sample Custodian who can then pass it on to the treating clinician.

The treating clinician must first determine whether the participant has explicitly consented to the feedback of the specific class of finding (whether pertinent or incidental) by referring to the original consent form. Difficulties may arise if the clinician is no longer treating the participant, or the participant has stopped attending the clinic.

#### Validation

Once it has been established that the participant has consented to feedback, the next stage of the pathway is activated. The validation stage within the framework provides for safeguards that ensure that the original research finding is verified, both clinically and analytically.^14^

The analytical validity of the research finding must be established by a CPA (Clinical Pathology Accreditation) laboratory, which will confirm the genetic variant in the particular participant. Findings are validated either by obtaining a fresh independent sample from the participant, or by using a pre-existing one (provided that it has solely been handled within a CPA setting) to confirm accuracy.^14^ However, before this, it is recommended that findings should be verified by the treating clinician in an additional experiment (for example, Sanger sequencing) in order to reduce the workload for the CPA laboratory.

Appropriately qualified experts work with the clinical researcher to assess the clinical utility and also the validity of the finding by evaluating the accuracy of the genetic variant in identifying or predicting the particular disease. Findings must be of significant clinical importance to the participant. Here a balancing act is carried out between the risks and the benefits of reporting back, given that there is always the danger of causing unnecessary harm to participants and their families by communicating findings. The benefit of informing participants of CSFs should considerably outweigh any potential harm that could be caused by reporting back.^14^

#### Feedback

Once the CSF has been validated, the treating clinician will then be able to feedback results to the individual participant if this is considered appropriate. Feedback will occur in the way the treating clinician considers most suitable. Given that the studies are all genetic in nature, the necessary expertise and protocols should be established to assist in reporting back. Final feedback therefore occurs via the original clinic, which obtained the initial samples from the participant. Where, on the other hand, the treating clinician decides in light of all the evidence (such as whether consent has been obtained) that it is not appropriate to feedback any CSFs and can justify this decision, the participant will not be informed.

The decision whether or not to feedback PFs and/or IFs for a given patient collection was taken by each Sample Custodian in consultation with their Research Ethics Committee, in light of the four key requirements for (i) explicit consent, (ii) significant clinical importance, (iii) analytical and clinical validity and (iv) the availability of an appropriate feedback mechanism, that were identified by the Ethical Advisory Group and were universally adopted across the UK10K project. The result of these deliberations was that none of the Sample Custodians undertook to feedback IFs, but almost all undertook to feedback PFs.

## The lifespan of the framework

The UK10K management framework was designed not only to provide a mechanism for dealing with CSFs discovered during the UK10K project, but it is hoped it will also provide a possible precedent that might be used in other projects. It provides for an effective flow of communication between researchers involved in the same research project but with different expertise based in different institutions. Information, that is relevant to both researchers and participants, is relayed from one researcher to another traversing the different stages, creating a cohesive framework, which ultimately allows for validated and clinically significant information to be returned, when appropriate and in a responsible way, to the participant in a clinical setting.

However, these stages of validation and assessment will not be possible to sustain once the UK10K project itself comes to an end. It is therefore intended that once the UK10K project is complete, the management framework will also come to an end. By this time, data will have been stored in the EGA and will remain accessible to researchers involved in other projects. Should these secondary researchers make any CSFs after the UK10K project has ended, it will no longer be possible for these to be passed on the UK10K Management Committee, as this will no longer exist. However, it might be possible for an appropriately constituted Data Access Committee to take on this role beyond the life-time of UK10K. Developing a governance mechanism for the management framework to be in place in the longer term needs further consideration by policy makers and funders, particularly as many sample collections will be continued to be studied after the UK10K project is completed.

## Conclusion

The UK10K framework addresses the principal ethical concerns when feeding back CSFs under specific conditions. Sample Custodians are obliged to pass on CSFs to the treating clinicians, who in turn are responsible for passing on CSFs to their patients where they meet the relevant criteria. It also ensures that those with no clinical role are not responsible for passing on any CSF they may find. It does, however, ensure that the procedure is there, should a CSF be identified. Assuming all of the criteria for feedback are met, the finding will pass through the management framework and the participant will be informed where appropriate. This may result in them receiving treatment, which may otherwise not have been prescribed.

However, it must be borne in mind that this approach does not address the important issue of IFs in population-based collections (where the issues of truly informed consent, and the provision of counselling remain poorly addressed as well as sequence data being of variable predictive capacity) and of course is a model likely to evolve and may need to be amended in response to changing circumstances. In the meantime, this framework brings us one step closer to bridging the gap between research and clinical care. The model has been tested with regards to PFs, where patients were informed before the first papers describing novel causal genes for rare diseases were published. The UK10K model establishes an important precedent for the feedback of CSFs in genetic and genomic research. It provides the foundation for a reflexive and responsive framework that will be able to address further logistical, ethical and legal issues as and when they come to light.
